# Development of Membrane Reactor Coupling Hydrogen and Syngas Production

**DOI:** 10.3390/membranes13070626

**Published:** 2023-06-28

**Authors:** Alexey A. Markov, Oleg V. Merkulov, Alexey Yu. Suntsov

**Affiliations:** 1 Institute of Solid State Chemistry and Mechanochemistry, Siberian Branch of the Russian Academy of Sciences (SB RAS), Novosibirsk 630090, Russia; aamarkov1@yandex.ru; 2 Institute of Solid State Chemistry, Ural Branch of the Russian Academy of Sciences (UB RAS), Yekaterinburg 620990, Russia; merkulov@ihim.uran.ru

**Keywords:** oxygen permeability, partial oxidation of methane, ceramic membrane, selectivity, synthesis gas, hydrogen production

## Abstract

Simultaneous syngas and pure hydrogen production through partial oxidation of methane and water splitting, respectively, were demonstrated by using mixed ionic–electronic conductors. Tubular ceramic membranes prepared from La_0.5_Sr_0.5_FeO_3_ perovskite were successfully utilized in a 10 M lab scale reactor by applying a radial arrangement. The supply of methane to the middle area of the reaction zone was shown to provide a uniform distribution of the chemical load along the tubes’ length. A steady flow of steam feeding the inner part of the membranes was used as oxidative media. A described configuration was found to be favorable to maintaining oxygen permeability values exceeding 1.1 mL∙cm^–2^∙min^–1^ and long-term stability of related functional characteristics. Methane’s partial oxidation reaction assisted by 10%Ni@Al_2_O_3_ catalyst proceeded with selectivity values above 90% and conversion of almost 100%. The transition from a laboratory model of a reactor operating on one tubular membrane to a ten-tube one resulted in no losses in the specific performance. The optimized supply of gaseous fuel opens up the possibility of scaling up the reaction zone and creating a promising prototype of a multitubular reaction zone with a simplified sealing procedure.

## 1. Introduction

There are some ecological issues becoming actual these days concerning industry processing. Among them, a number of harmful releases filling the atmosphere, especially carbon-containing gases, is distinguished as a general problem. In this regard, the widespread use of hydrogen as a main fuel is known to be appropriate for solving the abovementioned problem by decreasing the volume of unwanted carbon emissions [1,2,3,4]. Thus, hydrogen production becomes relevant, and therefore numerous methods were proposed [5,6,7,8,9]. At the same time, the use of electricity derived from renewable energy sources for hydrogen production was proved to be economically unprofitable [10,11]. In turn, natural gas is believed to be a more available and cheaper source of chemical energy. The main problem with its usage is the expensive separation of nitrogen from carbon dioxide, which accumulates during industrial processing. Additionally, hydrogen production directly from natural gas with the use of modern techniques is accompanied by some impurities of carbon-containing gases; therefore, an application of additional purification technologies is required that significantly elevates the hydrogen cost. A combination of the listed issues can be resolved by the careful implementation of separation technology using ceramic membranes based on mixed oxygen–electron conductors. Perfect gas density and excellent selectivity for oxygen ions exclude any admixtures of carbon-containing components in hydrogen produced. Carbon dioxide obtained after methane combustion doesn’t contain sufficient admixtures of inert gases that enables its safety utilization or future processing.

Membrane technology is considered here as an alternative one to known approaches enabling the simultaneous production of synthesis gas and hydrogen. One of them is superdiabatic combustion [12] involving H_2_/syngas production by hybrid porous medium catalyst reforming and by the use of biomass. Moreover, the use of thermochemical energy was demonstrated [13] to be effective for hydrogen and syngas production. This approach can be applied to a number of processes enabling coproduction procedures to take place; for instance, methane reforming by steam [14] or CO_2_ [15], hydrocarbon cracking [16], water thermolysis [17], etc. At the same time, it is obvious that solar energy can be considered as an auxiliary source. Moreover, most of the abovelisted processes require high temperatures. Chemical-looping steam methane reforming has been proposed as an effective route for providing syngas and hydrogen coproduction [18]. In this study, authors used perovskite-type oxides LaFe_1−x_Co_x_O_3_ as oxygen carriers which were used for methane oxidation, and steam was chosen as reducing media. Such configuration allows for the water splitting process and, therefore, hydrogen release. Nevertheless, the technology described only allows for achieving 85% methane conversion.

Last decade different groups proposed simple planar or single tube-type membrane reactors for partial methane oxidation on ceramic membranes [19,20,21,22,23]. Although the technologies described seem quite appropriate for laboratory experiments, their future application under the manufacturing scale requires a clear understanding of intrinsic processes being unspecific for lab-scale. We previously tested ceramic tube shape membranes based on La_0.5_Sr_0.5_FeO_3−δ_ oxide for the methane partial oxidation process [24,25,26]. Under the experiments made, methane flow was set along the membrane so that the lower part of the tube interacted with pure methane, whereas the upper one was in contact with the mixture of methane with partial oxidation products. As a result, the ceramic membrane suffered from uneven chemical load leading to rapid degradation of its bottom part. This feature should be noted, as the use of long tubular membranes is crucial, and conversely, can be neglected for small fragments. Moreover, such configuration was shown to contribute soot formation near feeding point, which generates from methane decomposition at low oxygen partial pressures and high temperatures. Soot deposition takes place mostly on the catalyst’s surface leading to granules crumbling and a build-up of pneumatic pressure inside the reaction zone. A reasonable approach to tackle this issue could be a maintaining of uniform distribution for methane flux as well as temperature over the membrane functional area. Such a solution is believed to ensure the implementation of membrane separation in a large-scale format and extend the life of the reactors.

The present work aimed to develop a lab-scale reactor with a radial arrangement of tubular membranes where the supply of natural gas (methane) is maintained continuously to the middle part of the reaction zone. Such configuration is found to be favorable for smooth distribution of chemical load along the tubes’ length as well as a uniform chemical expansion in the ceramic membranes used and, as a result, closed oxygen permeability and other functional characteristics.

## 2. Experimental Section

### 2.1. Synthesis and Membrane Formation

Complex oxide La_0.5_Sr_0.5_FeO_3−δ_ (LSF) was successfully synthesized via combustion of glycine-nitrate precursors obtained from high-purity starting reagents La_2_O_3_, SrCO_3_ and iron. Details of synthetic procedures and temperatures applied were reported previously [27]. The results of the X-ray powder diffraction (XRD) technique implemented with the Shimadzu-7000 (Kyoto, Japan) diffractometer were used to confirm the single-phase state of the obtained oxide. Ceramic membranes were formed by applying hydrostatic compression at 1 kbar, followed by annealing at 1300 °C. Thus, for the tests in a lab-scale reactor, gas-tight ceramic tubes with a length of 5 cm, diameter of 1 cm and wall thickness of 0.1 cm were made. Before the experiments, each membrane was checked for gas tightness by monitoring the leakage of air compressed at 1.5 atm from the surface of tubes immersed in ethyl alcohol.

### 2.2. Catalyst Preparation

The composite catalyst consisting of 10% Ni deposited on gamma alumina porous cylindrical granules with a diameter of 2 mm and length of up to 10 mm was used. NiO was chosen as the initial reagent with the required amount to obtain 10 wt % of metallic nickel in the final catalyst. A weighed oxide was entirely dissolved in nitric acid, and then aluminum oxide granules were impregnated by obtained nitrate solution. Uniform distribution of nickel over the granules’ surface was achieved by repeating the impregnation procedure four times involving intermediate calcinations at 400 °C. The obtained composites were then rapidly heated to 900 °C in order to form a nickel–aluminum spinel on the surface of the catalyst pellets. The obtained catalyst was checked by XRD. The specific surface area was measured by the BET method with the use of a Gemini VII (Micromeritics, Norcross, GA, USA) analyzer, which was 80 m^2^/g. Thermogravimetric measurements with the use of a TG92 (Setaram) analyzer were carried out in a hydrogen atmosphere to confirm the nickel content in the resulting catalyst.

### 2.3. Assembling of the Lab-Scale Reactor

A lab-scale reactor consisting of two functional areas was built from the Ni-Cr stainless steel analog to AISI321 grade. The first one includes a reaction zone where a corundum test tube vertically fixed in the middle was utilized for methane feeding through evenly spaced holes. Additionally, six steel tubes were placed symmetrically on the perimeter of the reaction zone to provide timely removal of reaction products. Moreover, six heating elements made from kanthal helix hermetically packed into quartz ampoules were evenly distributed to rule out a possible temperature gradient between the ceramic membranes.

**Figure 1:**
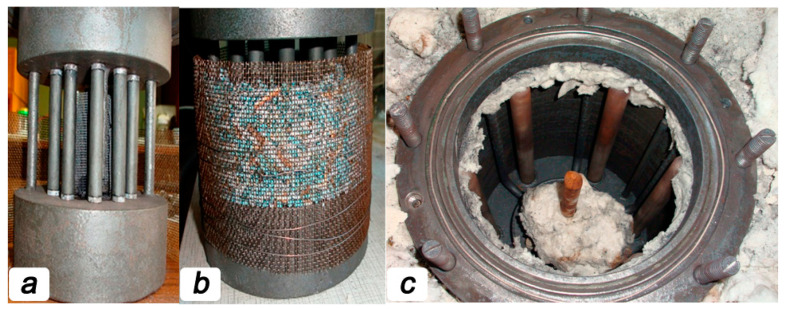
Images for the ceramic tubular membranes fixed in reaction zone before (**a**) and after (**b**) catalyst filling. Appearance of the reaction zone is presented in panel (**c**).

The rest of the reactor ensured a clamping of the oxygen-conducting membranes, keeping the catalyst in contact with the membranes and supplying steam into the membranes. In order to provide excellent gas tightness for the reaction zone, the membranes were sealed by high-temperature glass Schott No. 8252 (Germany) with a melting point of 950 °C. For sealing, rings 2 mm thick were cut from a glass tube of the appropriate size were glued to the ends of the membrane, which was then set in the reactor (Figure 1a). Then the catalyst was carefully poured on the outer side of the oxygen-conducting membranes so that the entire surface of the tubes was covered with granules. In this case, the thickness of the catalyst did not exceed 1 cm near the methane feeding point. A total of about 300 g of the catalyst was loaded into the reaction zone (Figure 1b).

In order to complete the reactor assembly, two prepared zones were conjoined, and then an outer contour of the reaction zone was sealed by using a silver ring sandwiched between two massive steel plates. The temperature of the reaction zone was changed with precision ±1 °C and maintained by Yokogawa UT-155 PID (TC) temperature controller (Tokyo, Japan). Additionally, S-type thermocouples were carefully fixed in the central part of the membrane and in a gas-tight steel shell located in the catalyst volume to analyze temperature distribution over the reaction zone.

**Figure 2:**
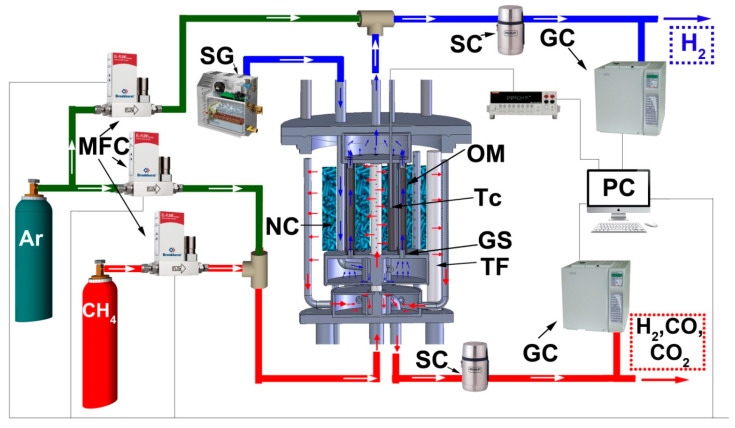
Schematic representation for the experimental reactor and auxiliary units for feeding control and measurements. MFC—mass flow controllers, SG—steam generator, SC—steam condenser, NC—nickel-containing catalyst, OM—oxide membrane, Tc—thermocouple, GS—glass sealant, TF—tubular furnace, GC—gas chromatograph, PC—personal computer.

Bronkhorst Fe201C (MFC) mass-flow controllers were utilized to set the flow values of the gases used. Steam feeding was maintained by a lab-made steam generator consisting of an electric heater placed into a dewar vessel half filled with water. The heater was governed by specially designed software, which provided the necessary steam flow in accordance with the pre-calibration results. Gas products were passed through steam condensers maintained at 0 °C for water separation. The composition of the outgoing gases was analyzed using a Crystal-5000 gas chromatograph (GC) with thermal conductivity detectors. The amount of hydrogen produced and its purity were periodically measured with a liquid displacement flow meter and a gas chromatograph, respectively. The detailed description of the reactor used and auxiliary measuring devices and feeding items is represented in Figure 2.

### 2.4. Reactor Warm-Up and Gas Feeding Peculiarities

In the first step reaction zone was heated up to a temperature sufficient for sealant glass softening. The heating rate was maintained at 5 °/min, and continuous feeding of outer and inside membrane surfaces by 40 mL/min Ar and 600 mL/min air was provided, respectively. The absence of nitrogen in the gas flow coming from the outer part of the membrane indicated that the sealing was complete; the reaction zone was then slowly cooled to working temperature, and airflow was gradually replaced by steam. The next step included careful admixing of methane flow equal to 15 mL/min to Ar flow and five hours of exposure for nickel catalyst activation. Furthermore, CH_4_ content in feeding media was smoothly increased to 100%, maintaining the total gas mixture rate. The procedure described, which takes about 20 h, is necessary to provide a smooth change in the oxygen activity in the gas phase on the membrane’s outer surface and to mitigate the mechanical stresses arising from the oxygen activity gradient across the wall of the ceramic tube. After the complete substitution of argon, the methane’s flow was slowly increased to the required value.

### 2.5. Preliminary Description of Ongoing Processes

The interaction between methane and the membrane surface leads to its partial or complete oxidation, as well as the formation of oxygen vacancies and electron charge carriers in the membrane material (1):

CH_4_ + 3[O^2–^] = CO_2_ + H_2_O + 3[V_O_] + 6ē (1)

Then, oxygen vacancies and electron charge carriers migrate to the opposite side of the membrane under the action of the chemical potential. Here, oxygen atoms from a water molecule are sorbed on the membrane surface and are incorporated into the oxygen vacancies of the complex oxide. In this case, the reduction in hydrogen atoms occurs with the formation of a hydrogen molecule (2)

H_2_O + [Vo] + 2ē = [O^2−^] + H_2_ (2)

The products obtained as a result of reaction (1) upon contact with the active centers of the catalyst and methane molecules interact according to the following reactions [28]

CO_2_ + CH_4_ = 2CO + 2H_2_ (3)

H_2_O + CH_4_ = CO + 3H_2_ (4)

It should be noted that active sites of nickel on the catalyst surface when interacting with methane, can form complexes of nickel carbide according to Equation (5): [29]

3Ni + CH_4_ = Ni_3_C + 2H_2_ (5)

In this case, if there are no oxygen-containing compounds in the surrounding atmosphere, the process of carbon formation proceeds, leading to the destruction of the catalyst granules. Additionally, in the presence of carbon dioxide and water vapor in the atmosphere, reactions (3) and (4) most likely proceed through the formation of nickel–carbon complexes on the catalyst surface.

### 2.6. Reactor Performance Assessment

During the experiments, gas flows forming in the reaction zone were analyzed by chromatography; thus, values for volume concentrations of gaseous products after partial methane oxidation were measured. The collected data were utilized to calculate methane conversion $X_{{\mathrm{CH}}_{4}}$, selectivity, *S*_CO_ and oxygen flux through ceramic membrane $F_{O_{2}}^{}$ according to respective Equations (1)–(3):

(6) $$X_{{\mathrm{CH}}_{4}}=\left(\frac{x_{\mathrm{CO}}+x_{{\mathrm{CO}}_{2}}}{x_{{\mathrm{CH}}_{4}}^{\mathrm{out}}+x_{\mathrm{CO}}+x_{{\mathrm{CO}}_{2}}}\right)\cdot 100\%$$

(7) $$S_{\mathrm{CO}}=\left(\frac{x_{\mathrm{CO}}}{x_{\mathrm{CO}}+x_{{\mathrm{CO}}_{2}}}\right)\cdot 100\%$$

(8) $$F_{O_{2}}^{}=F\cdot \left(2x_{{\mathrm{CO}}_{2}}+1.5x_{\mathrm{CO}}-0.5x_{H_{2}}-\frac{0.21}{0.78}\sqrt{\frac{28}{32}}\cdot x_{N_{2}}\right)$$

After the experiments, the state of the reaction zone was studied through visual inspection. The membrane’s microstructure was analyzed by scanning electron microscope (SEM) JEOL JSM 6390 (Tokyo, Japan) equipped with an energy-dispersive console used to determine atomic distributions over the surface.

## 3. Results and Discussion

### 3.1. Functional Characteristics of Partial Methane Oxidation

**Figure 3:**
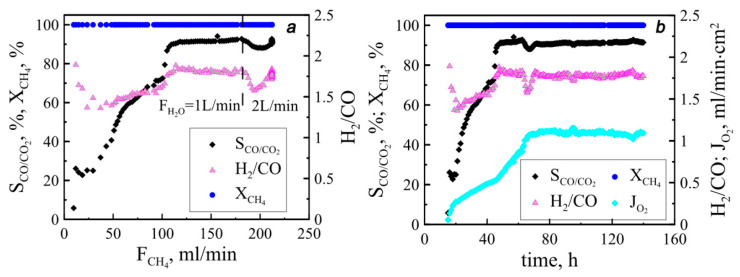
Functional parameters collected during experiments. Methane flow dependencies (**a**) correspond to warm-up step and time dependencies and (**b**) comply with operating conditions. Dashed line divides areas with different steam supply.

The results of reactor performance are presented in Figure 3. The value $X_{{\mathrm{CH}}_{4}}$ is seen to achieve almost 100%, which indicates high fuel conversion degree and an absence of catalyst-free spaces that permit unimpeded methane blowing. The methane oxidation process proceeding, in this case, is irreversible, i.e., methanation does not occur. The selectivity value of partial methane oxidation, indicating a part of the desired chemical reaction resulting from the reactor operation, is above 90%, which evidences the high performance of the catalyst used. In order to gain a deep understanding of the processes raised inside the reaction zone, the selectivity value should be compared with those obtained at different approaches applied for methane feeding. It should be emphasized here that a methane supply across the ceramic tube was used in this work so that the reducing gas was evenly distributed along the membrane. Therefore, the selectivity values of 90% achieved at 130 mL/min did not change with a further increase in flow methane. At the same time, the increase in feeding rate contributes to the oxygen permeability of the membranes. In the case of the longitudinal methane supply considered in the previous work [24], the selectivity increased gradually with methane concentration and reached high values only at the maximum membrane permeability. This difference confirms the previous assumption that under longitudinal methane supply, the ends of the membrane are in different conditions, and a subsequent increase in the methane flow leads to a gradual alignment of the chemical potential of oxygen along the membrane. In the case of transverse methane supply, all tubes are in practically the same conditions initially, and an increase in the methane flow leads to a uniform change in the chemical potential of oxygen over the membrane surfaces. It should be noticed that the oxygen permeability value reached over 140 mL/min from ten tubular membranes in the aggregate, which corresponds to a relative oxygen permeability of 1.1 mL/min∙cm^−2^.

**Figure 4:**
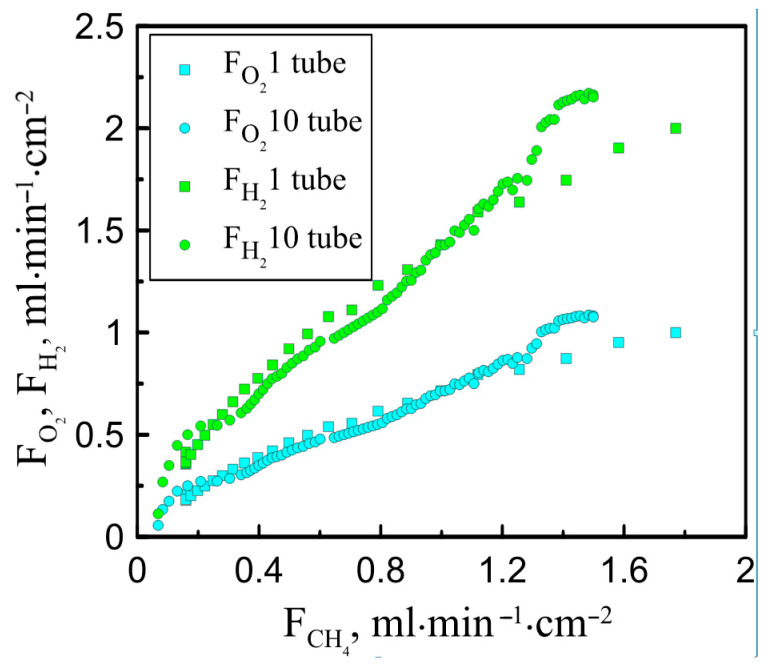
The experimentally measured values of hydrogen and oxygen fluxes obtained after water splitting and through ceramic membrane vs. methane flow feeding reaction zone.

Figure 4 presents a comparison of the obtained results with ones reported earlier for single-tube reactors [25]. It can be seen that the relative fluxes of oxygen and hydrogen are in good agreement for both reactors. However, at high values of the methane flux, a slight increase in the efficiency of the oxygen-conducting membranes is observed. It should be stressed that in this work, the steam supply per square centimeter of the membrane surface was set at 7 mL/min, and in the case of a single-tube reactor, it reached 26 mL/min∙cm^−2^. Increasing the steam flow to 14 mL/min∙cm^−2^ was found to lead to an increase in oxygen permeability, which also indicates a more efficient distribution of oxygen partial pressure when a ten-tube reactor is applied. It is also important to emphasize that the ratio of hydrogen to carbon monoxide does not exceed 2, which indicates the absence of soot formation in the reaction zone.

### 3.2. Study of Degradation Peculiarities

**Figure 5:**
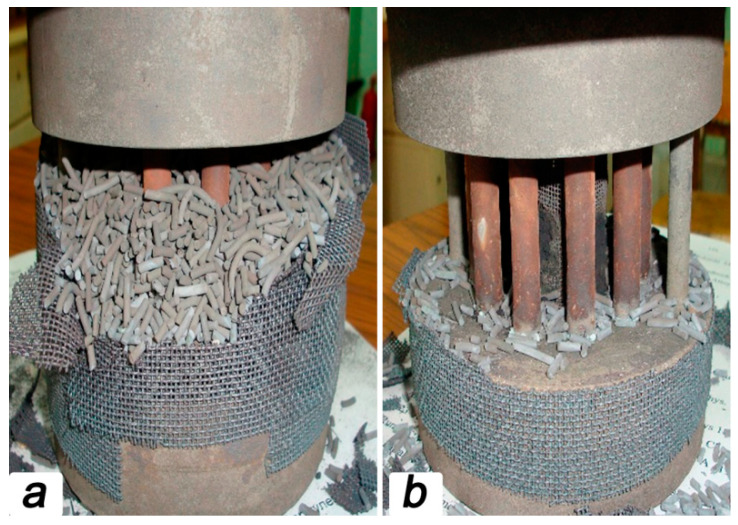
Appearance of the reaction zone containing catalyst (**a**) and only membrane module (**b**) after partial methane oxidation.

Figure 5 represents the appearance of the reaction zone still filled by catalyst (a) and partially cleaned (b) after the experiments. It should be noted that the catalyst granules, as well as ceramic membranes, retain the original shape and demonstrate excellent structural integrity. Moreover, it is important to highlight that the catalyst used does not evince any degradation signs associated with the soot formation.

**Figure 6:**
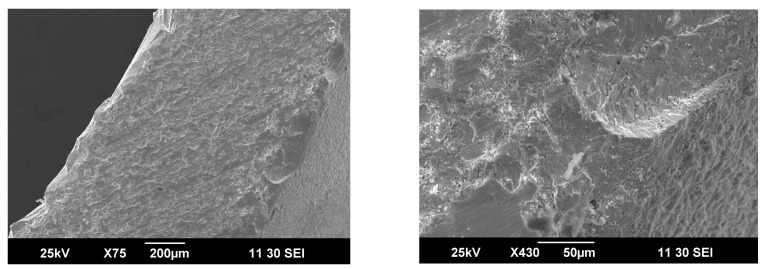
Cross-section images of the ceramic membranes obtained after partial oxidation tests.

In order to analyze membrane degradation peculiarities during the experiments, the sketch of the ceramic tube after treatment was carefully split and prepared for SEM analysis. As can be seen from the cross-sectional images depicted in Figure 6, the microstructure of ceramic remains mainly homogeneous, but some external layers on the outer side of the membranes can be distinguished. By taking into account the values of key functional parameters, which remain stable during the 140 h under experiment, it is seen that this barely noticeable layer is apparently preventing degradation of the membrane body.

**Figure 7:**
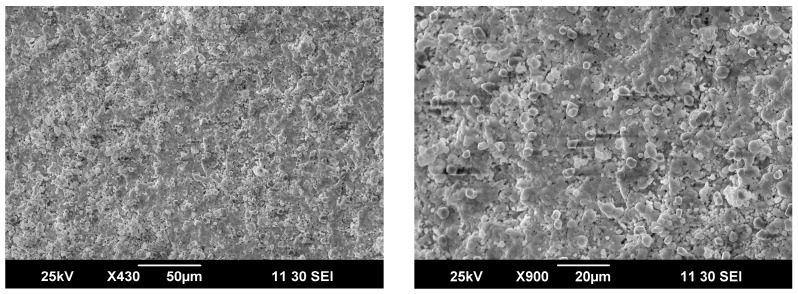
SEM images for outer surface of the ceramic membrane obtained after partial methane oxidation.

The outer surface of the membrane contacting with methane and the products of its partial oxidation is covered by small particles less than 5 μm, Figure 7. Furthermore, partial segregation of iron-rich particles was seen by analyzing respective energy dispersive spectra. Supposedly, these particles are formed under the action of a reducing media, but this process seems to be slow and does not have a significant impact on the partial oxidation process as well as on membrane degradation as a whole.

**Figure 8:**
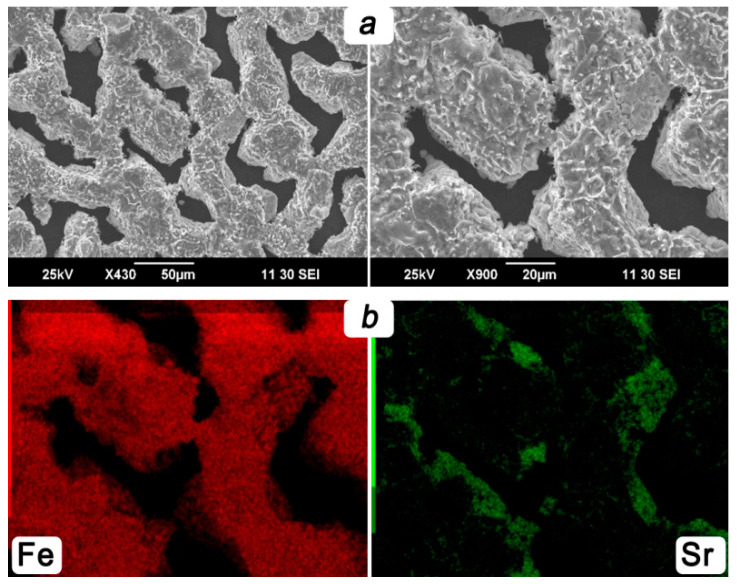
SEM images (**a**) and respective EDX maps (**b**) obtained from inner side of the ceramic membrane after steam exposure.

The analyzed SEM images obtained on the membrane’s inner side disclose interesting features. As can be seen from Figure 8a, prolonged exposure to steam on the ceramic membrane causes the formation of a thin metal mesh with a thickness of up to 10 μm and more than 20 μm in width. The results of the EDX analysis depicted in Figure 8b confirm that the covering layer almost consists of iron. It can be assumed that the observed layer can serve as a reinforcing unit, preventing the process of membrane destruction and contributing to a decrease in the energy barrier of water splitting. This is partially evidenced by stable parameters and the high efficiency of the reactor maintained for 140 h.

### 3.3. Future Prospects

**Figure 9:**
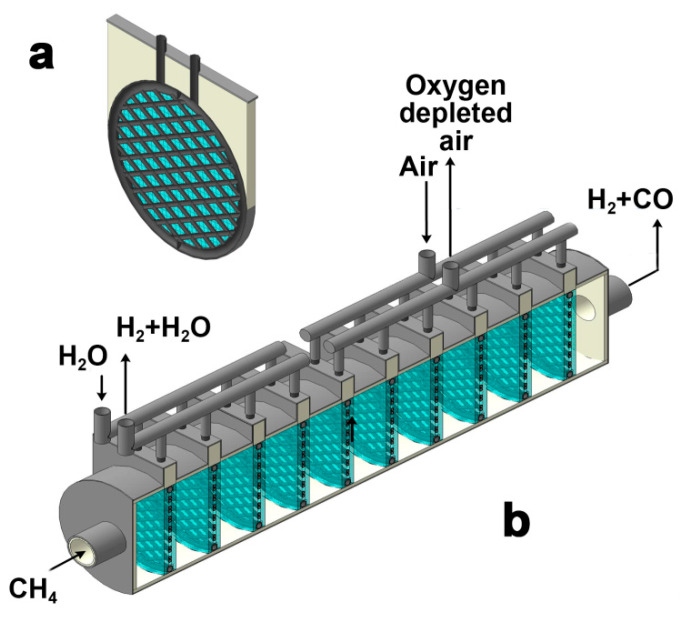
Single membrane module (**a**) and prototype of industrial-scale reactor (**b**) with simplified sealing configuration.

In order to implement the wide spreading of the demonstrated technology on an industrial scale, it is necessary to design reactor plants producing up to hundreds of cubic meters of hydrogen per hour. As a result, producing such amounts of hydrogen requires sealing hundreds or thousands of tubular ceramic membranes. In this case, the failure of even one functional element will lead to the shutdown of the membrane reactor as a whole. Obviously, such a configuration seems to be complex and, therefore, insufficient and inappropriate for industrial implementation. A possible way to simplify membrane sealing is the use of a cold zone with sealants based on organic polymers, but their stability is highly limited to 300 °C. Moreover, a significant elongation of tubular membranes leads to inefficient use of oxygen-conducting materials. Therefore, it is necessary to create more complex membranes forming a well-developed network of channels, which engage a more functional surface area of the membranes compared to the number of sealing joints. In this work, the concept developed is presented in Figure 9a. The proposed ceramic membrane consists of many tubular channels connected into a whole solid module to ensure a uniform distribution of gas flows. Such a single membrane element has one input and one output. Outside, the membrane is covered with a catalyst for the partial oxidation of methane fixed by a metal mesh.

The presented elements can be successively fixed in the tubular reactor, as demonstrated in Figure 9b. Such configuration allows one to combine the production of pure hydrogen from water steam with partial methane oxidation. It is important to note that the latter enables obtaining the heat, which might be expended for the first process. In addition, there is the possibility of joint utilization of membrane materials that are stable at various partial pressures of oxygen. Thus, the use of such reactor plants will make it possible to produce pure hydrogen and synthesis gas.

## 4. Conclusions

In summary, ten tubular ceramic membranes from La_0.5_Sr_0.5_FeO_3−δ_ were formed by isostatic pressure and then carefully sintered to a gas-tight state. The prepared membranes were sealed with gas-feeding channels and filled by NiO@Al_2_O_3_ composite catalyst at the outer side. After the warm-up procedure, the reaction zone was gradually fed by steam and methane to the inner and outer sides, respectively. The application of methane cross-feeding into the reaction zone instead of the longitudinal one studied previously provides a uniform fuel distribution over reaction volume and suppresses membrane degradation caused by irregular chemical potential set along the tube body. Functional parameters collected during experiments evidenced a deep fuel conversion achieving almost 100%, excellent combination of selectivity and relation of H_2_ to carbon monoxide. The calculated oxygen and hydrogen fluxes were shown to be comparable with those for single-tube reactors, but achieving these values requires smaller gas fuel flows. The study of membrane degradation revealed a formation of a mesh-like layer enriched by iron covering the inner side of the tube. The used membrane module was proposed for use in industrial-scale reactors, allowing for sealing in a cold state.

## Data Availability

Not applicable.
